# The association between chronic musculoskeletal pain and clinical outcome in chronic kidney disease patients: a prospective cohort study

**DOI:** 10.1080/0886022X.2019.1596817

**Published:** 2019-04-24

**Authors:** Heng-Jung Hsu, I-Wen Wu, Kuang-Hung Hsu, Chiao-Yin Sun, Ming-Jui Hung, Chun-Yu Chen, Chi-Jen Tsai, Mai-Szu Wu, Chin-Chan Lee

**Affiliations:** a Division of Nephrology, Chang Gung Memorial Hospital, Keelung, Taiwan;; b Taoyuan School of Medicine, the Graduate institute of Clinical Medical Sciences, Chang Gung University Medical College, Keelung, Taiwan;; c Laboratory of Epidemiology, Department of Health Care Management, Chang Gung University, Taipei, Taiwan;; d Division of Cardiology, Chang Gung Memorial Hospital, Keelung, Taiwan;; e Division of Nephrology, Taipei Medical University Hospital, Taipei, Taiwan;; f Department of Internal Medicine, Taipei Medical University, Taipei, Taiwan

**Keywords:** Chronic musculoskeletal pain, chronic kidney disease, progression, nonsteroidal anti-inflammatory drug, all-cause mortality

## Abstract

**Background and objectives**: Chronic musculoskeletal (MS) pain is common in chronic kidney disease (CKD) patients. The association of chronic MS pain and CKD progression has not yet been established.

**Method**: We conducted a prospective cohort study to evaluate the association of chronic MS pain and CKD progression of pre-dialysis CKD patients.

**Result**: A total of 53.2% of pre-dialysis CKD patients had chronic MS pain. Patients classified as progression and non-progression had a similar prevalence of chronic MS pain at baseline, and similar baseline use of NSAIDs and Chinese herbal medicines. Univariate Cox analysis indicated that chronic MS pain and baseline NSAID or Chinese herbal medicine use were not significantly associated with progression of CKD. But multivariate Cox regression found chronic MS pain was independently significantly associated with all-cause mortality (HR, 2.912, 95% CI, 1.004–8.444; *p* = .049). However, serum levels of hs-CRP were similar between those chronic MS pain patients and without chronic MS pain patients (4.96 ± 9.4 vs. 4.25 ± 13.3 mg/L, *p* = .535).

**Conclusion**: The CKD patients with chronic MS pain was independently and significantly associated with all-cause mortality, but not independently and significantly associated with CKD progression and composite endpoints. The inflammatory marker-hs-CRP was similar between CKD patients with and without chronic MS pain.

## Introduction

The incidence of chronic kidney disease (CKD) is still increasing worldwide despite integrative care and timely referral [1]. CKD patients had lots of complications which lead to high mortality [2]. Understanding the prognostic factor of CKD is important for planning strategy to prevent CKD progression.

Patients with CKD often suffer from chronic pain [3,4]. For example, a recent study reported that more than 50% of CKD patients experienced chronic pain, but less than 10% of non-CKD patients had chronic pain [3]. Musculoskeletal (MS) pain is the most common type of pain in CKD [3,5]. Other research has reported that a high percentage of CKD patients experience moderate-to-severe pain [6]. The possible cause of common chronic MS pain in CKD patients is multifactor. Co-morbidity of osteoarthritis, ischemic limb disease and also numerous painful syndromes unique to CKD, such as calciphylaxis and renal osteodystrophy all contribute to the higher prevalence of chronic MS pain in CKD patients [7].

Pain was considered as an important symptom in CKD patients [8,9]. In addition, perception of pain had a large impact on quality of life in CKD patients [10]. It had been shown that health-related quality of life was a predictor of mortality in CKD patients under hemodialysis [11]. Besides, chronic pain, especially severe chronic pain is associated with mortality in general population [12,13]. In advanced CKD patients, chronic MS pain is linked to inflammation [14]. Inflammation is one of the primary cause of CKD progression [15]. However, the relationship between chronic MS pain and clinical outcome in CKD patients is still unclear. That is why we conducted a prospective cohort study to evaluate the association between chronic MS pain and clinical outcome in CKD patients.

## Materials and methods

### Patients

Pre-dialysis CKD patients who visited an outpatient clinic at the Nephrology Department of Chang Gung Memorial Hospital at Keelung from March 2006 to July 2007 were recruited for the study. Patients satisfying the following criteria were included in this study consecutively: age >18–<80 years and with stable CKD in the 3 months before the start of the study. CKD was defined as the presence of persistent proteinuria or a decreased estimated glomerular filtration rate (eGFR) of <90 mL/min per 1.73 m^2^ [determined by the CKD Epidemiology Collaboration (CKD-EPI) creatinine equation] in 2 separate measurements within an interval of 3 months [16]. A total of 456 patients who provided informed consent were enrolled in the study. This study complies with the Declaration of Helsinki and was approved by the Ethics Committee of the Institutional Review Board (IRB number: 96-0407B) at Chang Gung Memorial Hospital. The study was conducted at the CKD center of the Chang Gung Memorial Hospital, Keelung, Taiwan.

### Study design

The study evaluated the association between chronic MS pain and renal prognosis in CKD patients. All eligible patients were carefully interviewed to identify primary disease and current medications. Chronic MS pain was evaluated by using the Community Oriented Program for the Control of Rheumatic Diseases (COPCORD) questionnaire [14]. All eligible patients were followed-up for about 6 years (up to 1 May 2012). During follow up, medical visits and renal function measurements were performed at 3, 6 and 12 months, and every 12 months thereafter until study primary endpoints (renal progression and/or death). A patient was classified as a ‘Renal progression’ if the eGFR was 50% or less of the baseline value, or if there was ESRD that required dialysis. A prospective cohort design was used to investigate the association of chronic MS pain and CKD progression.

For patients with MS pain, pain was assessed using a visual analog scale (VAS), on a range of 0 mm (no pain) to 100 mm (very painful), after which the patients were examined by a rheumatologist to confirm the information and characterize MS pain symptoms. For the calculation of prevalence and for all other analyses, chronic MS pain was defined as non-traumatic MS pain for over 3 months with a VAS score of >1 [9].

Basic demographic data were collected, including information on age, gender, nutrition status – body mass index (BMI), history of smoking, current use of NSAIDs or Chinese herbal medicines, and the presence of diabetes, hypertension, coronary artery disease (CAD), and hyperuricemia. Baseline hematological and biochemical data of these patients were also collected. Current use of NSAIDs was defined by self-reported use of ibuprofen, naproxen, sulindac, piroxicam, indomethacin, tolmetin, or diclofenac, COX (cyclooxygenase)-2 inhibitor (with brand names and combination formulas identified) daily or nearly every day for the last 30 days. Current use of Chinese herb was defined by self-reported use of any Chinese herb daily or nearly every day for the last 30 days. Full laboratory profiles were obtained. The laboratory parameters included levels of blood urea nitrogen, serum creatinine (Scr), hemoglobin, albumin, high-sensitivity C-reactive protein (hs-CRP), calcium (Ca) and phosphate (P). Serum creatinine levels were assessed by spectrophotometric analysis using a modified kinetic Jaffe reaction.

### Statistical analysis

Descriptive statistics are expressed as means ± standard deviations or as medians and ranges or percentages, as appropriate. Student’s *t*-test or the Mann–Whitney *U*-test was used to compare the means of continuous variables and the Chi-square test was used to compare categorical data. Kaplan–Meier curves were used to assess renal survival in patients. Adjusted risk estimates for progression to the endpoint were calculated using univariate regression, followed by multivariate Cox proportional hazard regression. Co-variants selection based on univariate analysis with variables with *p* < .05 was selected. All statistical tests were two-tailed, and a *p* value less than .05 was considered statistically significant. Data were analyzed using the SPSS 23.0 for Windows (SPSS Inc., Chicago, IL).

## Results

### Clinical and demographic characteristics of study population

The mean age was 63.3 ± 14.1 years and 194 patients (42.5%) were male (Table 1). The mean eGFR was 65 ± 34 mL/min/1.73 m^2^. A total of 243 patients (53.3%) had chronic MS pain. At baseline, 36 patients (7.8%) reported the use of an NSAID and 44 patients (9.6%) reported the use of a Chinese herbal medicine. Those patients with male, higher BMI, co-morbidity of hyperuricemia, higher levels of systolic blood pressure, lower levels of proteinuria, higher serum levels of hemoglobin and product of serum calcium and phosphate levels, and less co-morbidity of diabetes-prone to associated with chronic MS pain. In our study patients, the prevalence of mild pain, moderate pain and severe pain are 15.6% (*n* = 38/243), 28.4% (*n* = 69/243) and 58% (*n* = 141/243), respectively. About the CKD stage and chronic MS pain severity, severe chronic MS pain was found in 54.2% (*n* = 64/118) of CKD patients with stage 1–2 disease, 54.9% (*n* = 62/113) of CKD patients with stage 3–4 disease and 83.3% (*n* = 10/12) of CKD patients with stage 5 disease (*p* < .001). About the cause of chronic MS pain, we found 71% of those chronic MS pain had co-morbidity of gout, 0.4% had co-morbidity of rheumatoid arthritis, 1.2% had co-morbidity of spine osteoarthritis, 19.8% had co-morbidity of back pain. In our study with follow-up of about 6 years, there were 13 patients drop out, 31 patients died, 36 patients initiated hemodialysis, 10 patients initiated peritoneal dialysis at the end of the study.

**Table 1.** Baseline characteristics and laboratory parameters in chronic kidney disease according to the presence of chronic musculoskeletal (MS) pain.

	All patients n = 456	No chronic MS pain n = 213	Chronic MS pain n = 243	p
Age (y)	63.3 ± 14.1	62.4 ± 14.5	64.6 ± 13.5	.09
Male (*n*, %)	194 (42.5%)	64 (30.0%)	130 (53.5%)	<.001*
Body mass index (Kg/m^2^)	25.5 ± 3.9	25.0 ± 3.4	26.3 ± 4.3	.001*
Smoking (*n*, %)	181 (39.7%)	89 (41.8%)	92 (37.8%)	.754
NSAID use (*n*, %)	36 (7.8%)	16 (7.5%)	20 (8.2%)	.854
Chinese herb use	44 (9.6%)	22 (10.3%)	22 (9.1%)	.822
Use of ACEI/ARB (*n*, %)	370 (81.1%)	180 (84.5%)	190 (78.2%)	.713
Co-morbidity				
Diabetes (*n*, %)	140 (30.7%)	75 (35.2%)	65 (26.7%)	.034*
Hypertension (*n*, %)	286 (62.7%)	124 (58.2%)	162 (66.7%)	.059
CAD (*n*, %)	35 (7.6%)	19 (8.9%)	16 (6.5%)	.312
Hyperuricemia (*n*, %)	226 (49.6%)	54 (25.5%)	172 (71.0%)	<.001*
CKD stage				.074
1 (*n*, %)	129 (28.3%)	77 (36.2%)	52 (21.4%)	
2 (*n*, %)	110 (24.1%)	44 (20.7%)	66 (27.2%)	
3 (*n*, %)	113 (24.8%)	49 (23.0%)	64 (26.3%)	
4 (*n*, %)	81 (17.8%)	32 (15.0%)	49 (20.2%)	
5 (*n*, %)	23 (5.0%)	11 (5.2%)	12 (4.9%)	
Blood pressure				
Systolic pressure (mmHg)	133 ± 19	130 ± 18	135 ± 19	.006*
Diastolic pressure (mmHg)	73 ± 10	72 ± 10	74 ± 11	.631
Proteinuria (mg/day)	672.8 ± 1463.6	825.7 ± 1781.2	472.6 ± 855.2	<.001*
Initial laboratory finding				
BUN (mg/dL)	8.4 ± 3.4	8.6 ± 3.6	8.3 ± 3.1	.019
Scr (mg/dL)	1.5 ± 1.1	1.4 ± 1.1	1. 6 ± 1.1	.119
eGFR (mL/min/1.73 m^2^)	65 ± 34	68 ± 36	63 ± 31	.107
Hemoglobin (g/dL)	11.8 ± 2.1	11.4 ± 2.1	12.4 ± 2.1	.010*
Albumin (g/dL)	3.9 ± 0.5	3.9 ± 0.6	3.9 ± 0.5	.882
hsCRP (mg/L)	4.6 ± 11.8	4.25 ± 13.3	4.96 ± 9.4	.535
Ca (mg/dL)	9.2 ± 0.7	9.3 ± 0.5	9.2 ± 0.8	.631
P (mg/dL)	3.9 ± 0.8	3.8 ± 0.7	4.0 ± 0.9	.222
Ca × P (mg^2^/mL^2^)	32.3 ± 13.4	30.7 ± 13.5	34.1 ± 13.3	.039*

*p < .05. BMI: body mass index; NSAID: nonsteroidal anti-inflammatory drug; ACEI: angiotensin converting enzyme inhibitor; ARB: angiotensin receptor blockage; eGFR: estimated glomerular filtration rate; CAD: coronary artery disease; CKD: chronic kidney disease; BUN: blood urea nitrogen; Scr: serum creatinine; hs-CRP: high sensitive-C reactive protein; Ca: calcium; P: phosphate; Ca × P: the product of calcium and phosphate; MS: musculoskeletal.

### Factors associated with renal progression

**Figure 1.**
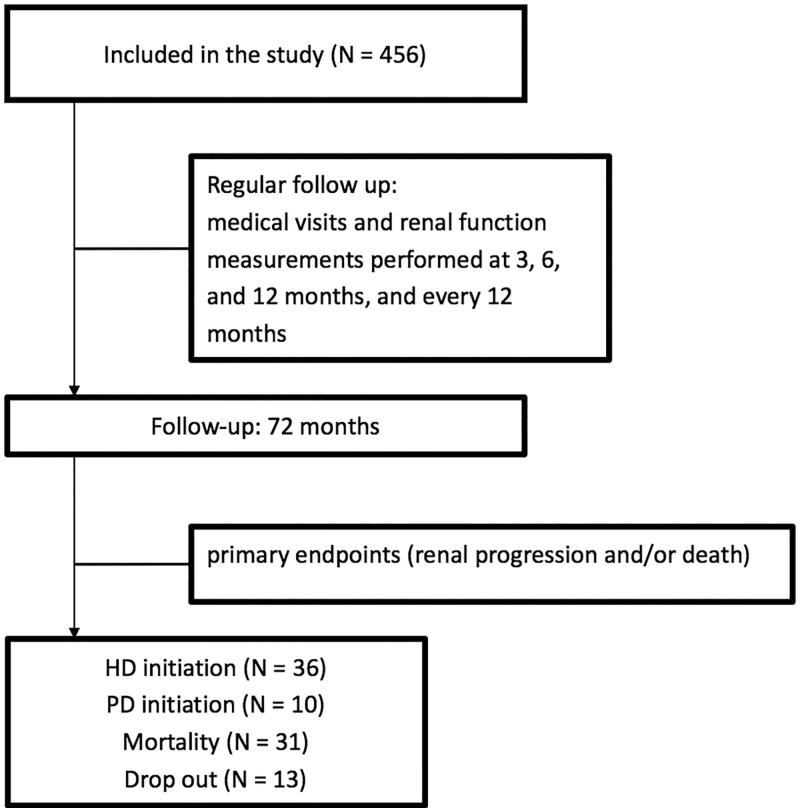
Enrollment scheme and patient status. HD: hemodialysis; PD: peritoneal dialysis.

A total of 82 patients (17.9%) experienced renal progression with a mean follow-up time of 44.6 ± 12.8 months. Among these 82 patients, 36 patients had eGFR less than 50% of baseline and 46 patients progressed to ESRD (Figure 1). Patients classified as progression and non-progression were significantly different in numerous demographic and clinical variables (Table 2). However, these two groups had a similar baseline prevalence of chronic MS pain (42 [51.2%] vs. 201 [53.7%], *p* = .857), use of NSAID (8 [9.7%] vs. 28 [7.4%], *p* = .114), and use of a Chinese herbal medicine (10 [12.2%] vs. 34 [9.1%], *p* = .721).

**Table 2.** Baseline characteristics and laboratory parameters in study CKD patients with renal progression or not.

	All patients	Progression	Non-progression	*p*
	*n* = 456	*n* = 82	*n* = 374	
Age, years	64.4 ± 14.3	71.5 ± 12.3	63.2 ± 14.2	<.001
Male, *n* (%)	194 (42.5%)	30 (36.5%)	164 (43.8%)	.061
Body mass index, kg/m^2^	25.1 ± 4.0	24.5 ± 4.2	25.2 ± 4.0	<.001
NSAID use, *n* (%)	36 (7.8%)	8(9.7%)	28 (7.4%)	.114
Chinese Herb use, *n* (%)	44 (9.6%)	10 (12.2%)	34 (9.1%)	.721
Use of ACEI/ARB (*n*, %)	370 (81.1%)	61 (74.3%)	309 (82.6%)	.713
Diabetes, *n* (%)	140 (30.7%)	39 (47.6%)	101(27.0%)	<.001
^$^eGFR, mL/min/1.73 m^2^	55 ± 38	19 ± 17	60 ± 38	<.001
Initial CKD stage				<.001
1, *n* (%)	129 (28.3%)	6 (7.3%)	123(32.8%)	
2, *n* (%)	110 (24.1%)	6(7.3%)	104 (27.8%)	
3, *n* (%)	113 (24.8%)	19 (23.1%)	94 (25.1%)	
4, *n* (%)	81 (17.8%)	33 (40.2%)	48 (12.8%)	
5, *n* (%)	23 (5.0%)	14 (17.1%)	9 (2.4%)	
Hemoglobin, g/dL	10.9 ± 2.2	9.7 ± 1.9	11.4 ± 2.2	<.001
Albumin, g/dL	3.8 ± 0.7	3.4 ± 0.7	3.9 ± 0.6	<.001
hs-CRP, mg/L	3.2 (0.2–48.4)	3.27 (0–195)	1.20 (0–188)	<.001
Ca, mg/dL	8.9 ± 0.8	8.6 ± 1.0	9.0 ± 0.7	<.001
P, mg/dL	4.5 ± 1.3	4.9 ± 1.5	4.2 ± 1.1	<.001
Ca × P, mg^2^/dL^2^	38.4 ± 44.3	40.8 ± 13.7	37.1 ± 9.4	.001
Uric acid, mg/dL	5.5 ± 2.1	6.9 ± 2.0	5.3 ± 2.0	<.001
Systolic blood pressure, mmHg	133 ± 19	132 ± 20	142 ± 22	<.001
Diastolic blood pressure, mmHg	73 ± 10	74 ± 11	74 ± 12	.52
Proteinuria	672.8 ± 1463.6	380.7 ± 876.5	2673.4 ± 2657.5	<.001
Chronic MS pain, *n* (%)	243 (53.2%)	42 (51.2%)	201(53.7%)	.857

NSAID: nonsteroidal anti-inflammatory drug; ACEI: angiotensin converting enzyme inhibitor; ARB: angiotensin receptor blockage; eGFR: estimated glomerular filtration rate; CKD: chronic kidney disease; hs-CRP: high sensitive-C reactive protein; Ca: calcium; P: phosphate; Ca × P: the product of calcium and phosphate; MS: musculoskeletal.

$ The eGFR is the baseline eGFR.

### Factors associated with primary and composite endpoints by univariate Cox regression

**Figure 2.**
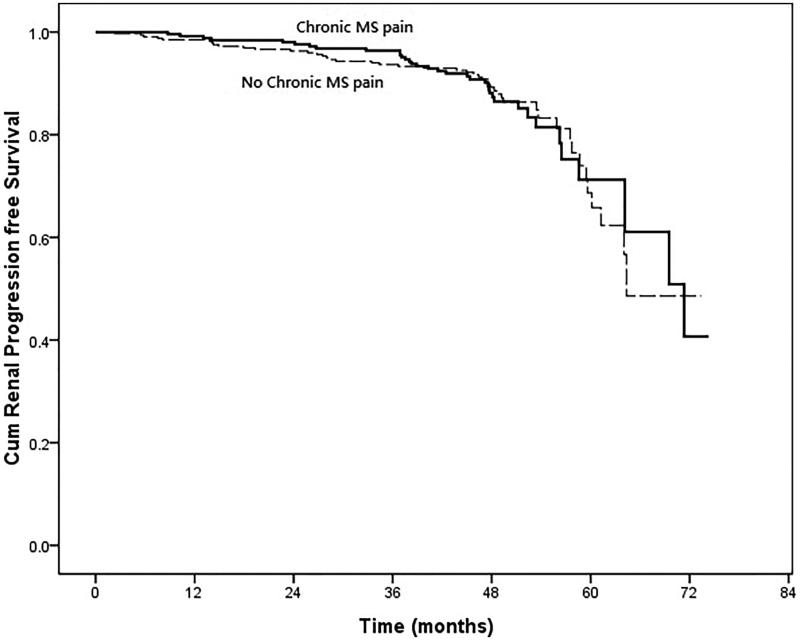
Cumulative proportion of patients who did not reach renal progression [defined as reduction of eGFR by 50% or end-stage renal disease (ESRD) requiring dialysis] censored for death in CKD patients with chronic musculoskeletal (MS) pain and without MS pain. (Cox–Mantel log rank test, *p* = .983).

**Figure 3.**
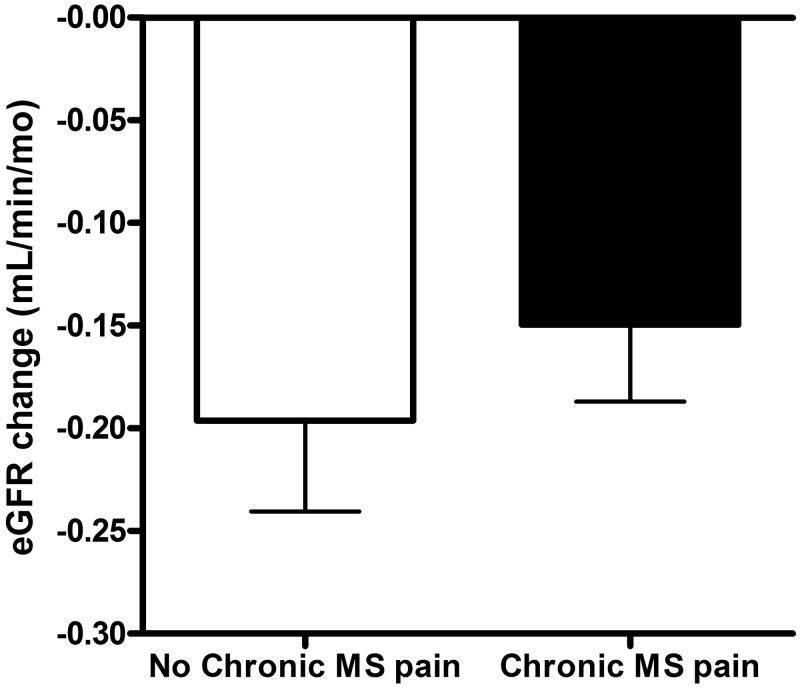
Change of eGFR between the CKD patients with chronic musculoskeletal (MS) pain and without MS pain (chronic MS pain vs. no chronic MS pain: −0.19 ± 0.08 vs. −0.15 ± 0.06 mL/min/mo, *p* = .446).

**Figure 4.**
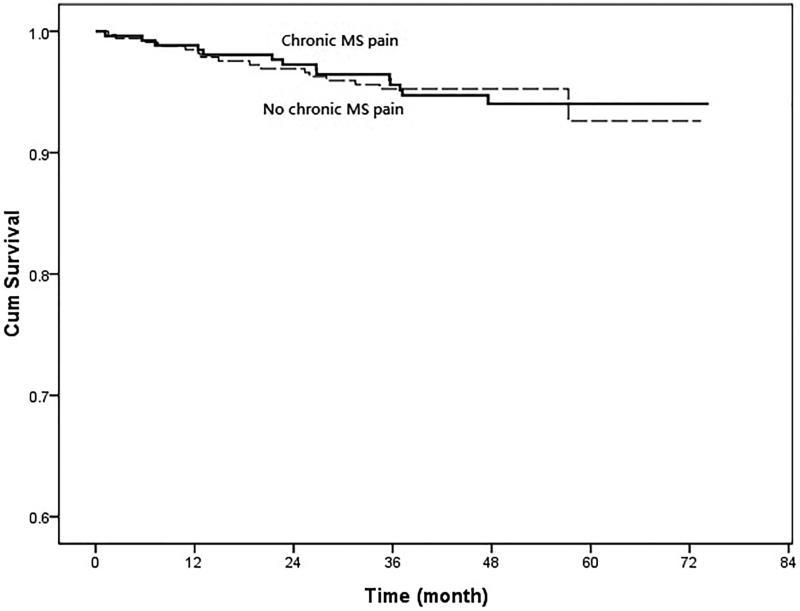
Cumulative survival curves of patients with chronic musculoskeletal (MS) pain and without MS pain. (Cox–Mantel log rank test, *p* = .774).

**Figure 5.**
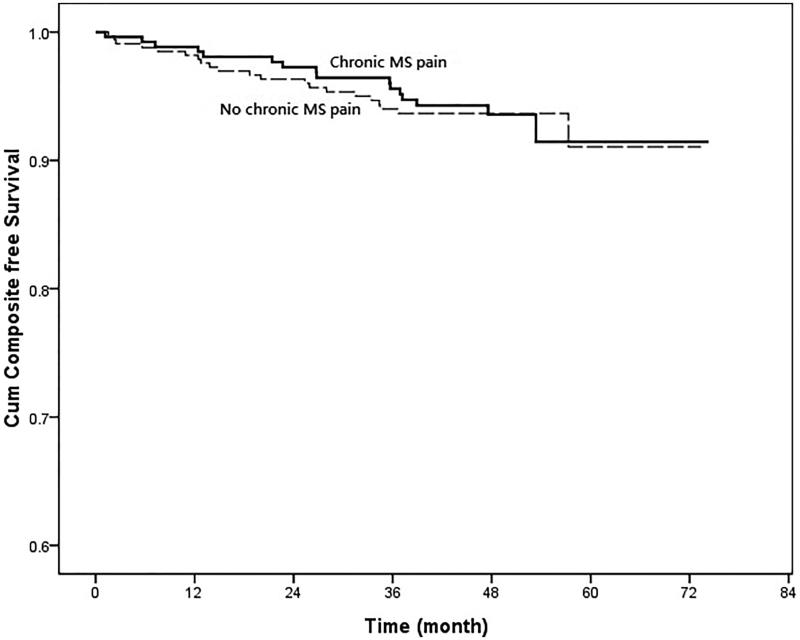
Cumulative proportion of patients who did not reach renal progression [defined as reduction of eGFR by 50% or end-stage renal disease (ESRD) requiring dialysis] or mortality in CKD patients with chronic musculoskeletal (MS) pain and without MS pain. (Cox–Mantel log rank test, *p* = .983).

In the univariate Cox regression, those patients with co-morbidity of diabetes (HR, 6.155; 95% CI, 3.819–9.921; *p* < .001), hypertension (HR, 2.541; 95% CI, 1.466–4.406; *p* = .001), lower eGFR (HR, 0.965; 95% CI, 0.955–0.975; *p* < .001), low serum levels of hemoglobin (HR, 0.845; 95% CI, 0.744–0.960; *p* = .010), low serum levels of albumin (HR, 0.511; 95% CI, 0.3240–0.806; *p* = .004), low serum levels of calcium (HR, 0.374; 95% CI, 0.199–0.702; *p* = .002), high serum levels of phosphate (HR, 2.985; 95% CI, 1.903–4.683; *p* < .001), high product of serum levels of calcium and phosphate (HR, 1.057; 95% CI, 1.015–1.101; *p* = .008), higher serum levels of uric acid (HR, 1.207; 95% CI, 1.095–1.329; *p* < .001), higher systolic blood pressure (HR, 1.021; 95% CI, 1.017–1.025; *p* = .008), higher levels of proteinuria (HR, 1.445; 95% CI, 1.344–1.554; *p* < .001) were significantly associated with renal progression (Table 3). However, those patients with chronic MS pain were not significantly associated with renal progression (HR, 1.004; 95% CI, 0.649–1.555; *p* = .984) (Figure 2). The eGFR changes during follow up were also not significant between those CKD patients with and without chronic MS pain (−0.15 ± 0.06 vs. −0.19 ± 0.08 mL/min/mo, *p* = .446) (Figure 3). About all-cause mortality, those patients with old age (HR, 1.103; 95% CI, 1.060–1.148; *p* < .001), low BMI (HR, 0.855; 95% CI, 0.760–0.962; *p* = .009), low levels of eGFR (HR, 0.979; 95% CI, 0.966–0.991; *p* = .001), high levels of hs-CRP (HR, 1.107; 95% CI, 1.007–1.027; *p* = .001), high levels of uric acid (HR, 1.303; 95% CI, 1.127–1.507; *p* < .001), low levels of diastolic blood pressure (HR, 0.956; 95% CI, 0.943–0.969; *p* < .001), higher levels of proteinuria (HR, 1.264; 95% CI, 1.082–1.477; *p* = .003) were significantly associated with all-cause mortality. But those CKD patients with chronic MS pain were not significantly associated with higher all-cause mortality (HR, 1.045; 95% CI, 0.515–2.120; *p* = .903) (Figure 4). In addition, those patients with co-morbidity of diabetes (HR, 2.773; 95% CI, 1.822–4.221; *p* < .001), low eGFR (HR, 0.986; 95% CI, 0.976–0.997; *p* = .013), low serum levels of hemoglobin (HR, 0.855; 95% CI, 0.752–0.971; *p* = .016), low serum levels of albumin (HR, 0.445; 95% CI, 0.275–0.719; *p* = .001), high serum levels of hs-CRP (HR, 1.014; 95% CI, 1.002–1.026; *p* = .022), low serum levels of calcium (HR, 0.423; 95% CI, 0.236–0.758; *p* = .004), high serum levels of phosphate (HR, 2.143; 95% CI, 1.385–3.316; *p* = .001), high serum levels of uric acid (HR, 1.314; 95% CI, 1.152–1.499; *p* < .001), high levels of systolic blood pressure (HR, 1.014; 95% CI, 1.009–1.018; *p* < .001), low levels of diastolic blood pressure (HR, 0.979; 95% CI, 0.970–0.988; *p* < .001), and higher levels of proteinuria (HR, 1.290; 95% CI, 1.13–1.472; *p* < .001) were significantly associated with composite endpoints. Furthermore, those CKD patients with chronic MS pain were not significantly associated with composite endpoints (HR, 1.054; 95% CI, 0.710–1.565; *p* = .774) (Figure 5). In addition, the patients with NSAID or Chinese herbal medicine use were not associated with renal progression, all-cause mortality or composite endpoints.

**Table 3.** Unadjusted hazard ratios (HRs) for primary and composite endpoints.

Baseline variable	Units of increase	Renal progression (event/total = 82/331)		All-cause mortality (event/total = 31/331)		Composite endpoint (event/total = 100/331)	
		Unadjusted HR (95% CI)	*p*	Unadjusted HR (95% CI)	*p*	Unadjusted HR (95% CI)	*p*
Age	1 year	1.005 (0.988–1.022)	.575	1.103 (1.060–1.148)	<.001	0.990 (0.974–1.007)	.258
Male vs. female	–	0.728 (0.471–1.125)	.153	1.613 (0.759–3.426)	.213	0.700 (0.469–1.046)	.082
Body mass index	1 Kg/m^2^	1.013 (0.954–1.076)	.666	0.855 (0.760–0.962)	.009	0.981 (0.923–1.043)	.548
NSAID usage (yes vs. no)	–	0.580 (0.231–1.455)	.246	0.491 (0.066–3.667)	.488	0.813 (0.191–3.471)	.780
Chinese herb usage (yes vs. no)	–	1.095 (0.522–2.297)	.810	1.949 (0.663–5.731)	.226	1.083 (0.557–2.104)	.814
Use of ACEI/ARB (yes vs. no)	–	1.214 (0.555–2.655)	.627	0.713 (0.178–2.860)	.633	0.481(0.166–1.388)	.176
Diabetes (yes vs. no)	–	6.155 (3.819–9.921)	<.001	1.871 (0.917–3.821)	.085	2.773 (1.822–4.221)	<.001
hypertension (yes vs. no)	–	2.541(1.466–4.406)	.001	1.186 (0.567–2.482)	.650	1.196 (0.736–1.944)	.470
eGFR	1 mL/min/1.73 m^2^	0.965 (0.955–0.975)	<.001	0.979 (0.966–0.991)	.001	0.986 (0.976–0.997)	.013
Hemoglobin	1 g/dL	0.845 (0.744–0.960)	.010	0.894 (0.699–1.144)	.374	0.855 (0.752–0.971)	.016
Albumin	1 g/dL	0.511 (0.324–0.806)	.004	0.597 (0.263–1.352)	.216	0.445 (0.275–0.719)	.001
hs-CRP	1 mg/L	1.000 (0.983–1.018)	.964	1.017 (1.007–1.027)	.001	1.014(1.002–1.026)	.022
Ca	1 mg/dL	0.374 (0.199–0.702)	.002	1.082 (0.478–2.448)	.850	0.423 (0.236–0.758)	.004
P	1 mg/dL	2.985 (1.903–4.683)	<.001	0.856 (0.368–1.991)	.718	2.143 (1.385–3.316)	.001
Ca × P	1 mg2/dL^2^	1.057 (1.015–1.101)	.008	0.981 (0.932–1.033)	.465	1.022 (0.986–1.059)	.230
Uric acid	1 mg/dL	1.207 (1.095–1.329)	<.001	1.303 (1.127–1.507)	<.001	1.314 (1.152–1.499)	<.001
Systolic blood pressure	1 mmHg	1.021 (1.017–1.025)	<.001	1.006 (0.999–1.012)	.095	1.014 (1.009–1.018)	<.001
Diastolic blood pressure, mmHg	1 mmHg	1.017 (0.999–1.015)	.109	0.956 (0.943–0.969)	<.001	0.979 (0.970–0.988)	<.001
Proteinuria	g/day	1.445 (1.344–1.554)	<.001	1.264 (1.082–1.477)	.003	1.290 (1.13–1.472)	<.001
Chronic MS pain (yes vs. no)	–	1.004 (0.649–1.555)	.984	1.045 (0.515–2.120)	.903	1.054 (0.710–1.565)	.774

Composite endpoint was defined as renal progression or all-cause mortality.

NSAID: nonsteroidal anti-inflammatory drug; ACEI: angiotensin converting enzyme inhibitor; ARB: angiotensin receptor blockage; eGFR: estimated glomerular filtration rate; hs-CRP: high sensitive-C reactive protein; Ca: calcium; P: phosphate; Ca × P: the product of calcium and phosphate; MS: musculoskeletal; BMI: body mass index.

### Adjusted HRs for primary and composite endpoints by multivariate Cox regression analysis

The analysis of multivariate Cox regression was also performed to evaluate the exact association between chronic MS pain and other factors, and primary and composite endpoints. The results indicated that the presence of chronic MS pain was not independently significantly associated with renal progression (HR, 1.428, 95% CI, 0.795–2.564; *p* = .233) and composite endpoints (HR, 0.917, 95% CI, 0.878–6.161; *p* = .089) (Table 4). But the presence of chronic MS pain was independently significantly associated with all-cause mortality (HR, 2.912, 95% CI, 1.004–8.444; *p* = .049) after adjustment of age, gender, co-morbidity of diabetes, hypertension, baseline eGFR, serum hemoglobin, albumin, hs-CRP, calcium, phosphate, Ca × P, and proteinuria. Besides, co-morbidity of diabetes mellitus (HR, 3.675, 95% CI, 1.975–6.835; *p* < .001) and proteinuria (HR, 1.387, 95% CI, 1.259–1.528; *p* < .0016) were also significantly independently associated with renal progression. And proteinuria was independently significantly associated with composite endpoints (HR, 1.285, 95% CI, 1.031–1.600; *p* = .026). However, baseline NSAID or Chinese herbal medicine use was also not independently significantly associated with primary and composite endpoints. The study also showed increased uric acid level is significantly associated with renal progression, all-cause mortality, and composite endpoints (renal progression and/or mortality). But the association between uric acid level and renal progression, all-cause mortality and composite endpoints (renal progression and/or mortality) loss significance after adjustment in the multivariate cox-regression analysis.

**Table 4.** Multivariate cox regression analysis for primary and composite endpoints.

Model	Renal progression (event/total = 82/331)			All-cause mortality (event/total = 31/331)			Composite endpoint (event/total = 100/331)		
	HR	95% CI	*p*	HR	95% CI	*p*	HR	95% CI	*p*
Diabetes mellitus (yes vs. no)									
unadjusted	6.155	3.819–9.921	<.001	1.871	0.917–3.821	.085	2.773	1.822–4.221	<.001
#adjusted	3.675	1.975–6.835	<.001	1.609	0.539–4.808	.394	1.986	0.732–5.390	.178
Hypertension (yes vs. no)									
unadjusted	2.541	1.466–4.406	.001	1.186	0.567–2.482	.65	1.196	0.736–1.944	.47
#adjusted	1.343	0.658–2.741	.418	1.233	0.435–3.494	.694	1.403	0.527–3.737	.498
Hs-CRP (1 mg/L)									
unadjusted	1.000	0.983–1.018	.964	1.017	1.007–1.027	.001	1.014	1.002–1.026	.022
#adjusted	0.997	0.967–1.028	.838	1.007	0.974–1.042	.663	1.006	0.973–1.040	.734
Uric acid (1 mg/L)									
unadjusted	1.207	1.095–1.329	<.001	1.303	1.127–1.507	<.001	1.314	1.152–1.499	<.001
#adjusted	0.962	0.765–1.209	.739	1.419	0.808–1.633	.439	1.130	0.817–1.562	.461
Proteinuria (1 g/day)									
unadjusted	1.445	1.344–1.554	<.001	1.264	1.082–1.477	.003	1.290	1.130–1.472	<.001
#adjusted	1.387	1.259–1.528	<.001	1.264	0.975–1.638	.077	1.285	1.031–1.600	.026
Chronic MS pain (yes vs. no)									
unadjusted	1.004	0.649–1.555	.984	1.045	0.515–2.120	.903	1.054	0.710–1.565	.794
#adjusted	1.428	0.795–2.564	.233	2.912	1.004–8.444	.049	2.326	0.878–6.161	.089
NSAID usage (yes vs. no)									
unadjusted	0.580	0.231–1.455	.246	0.491	0.066–3.667	.488	0.813	0.191–3.471	.780
#adjusted	0.736	0.218–2.481	.621	0.473	0.061–3.671	.474	0.862	0.191–3.900	.847
Chinese Herb usage (yes vs. no)									
unadjusted	1.095	0.522–2.297	.810	1.949	0.663–5.731	.226	1.083	0.557–2.104	.814
#adjusted	0.752	0.270–2.092	.585	2.368	0.625–8.972	.205	1.685	0.459–6.180	.431

#adjusted for age (1-year increment), male gender, diabetes status, hypertension status, eGFR (1 mL/min increments), serum hemoglobin (1 g/dL increments), albumin (1 g/L increments), hs-CRP (1 mg/L increments), calcium (1 mg/dL increments), phosphate (1 mg/dL increments), Ca × P (1 mg^2^/dL2 increments), proteinuria (1 g/day).

MS: musculoskeletal; NSAID: nonsteroidal anti-inflammatory drug.

## Discussion

Numerous factors underlie the progression of CKD. The present study enrolled pre-dialysis CKD patients and evaluated the effect of chronic MS pain on CKD progression. The present study was a prospective follow-up study of patients and considered numerous measurable risk factors for CKD progression, including the presence of chronic MS pain, use of an NSAID, and use of a Chinese herbal medicine. The main results found that those patients with and without chronic MS pain had similar risk for CKD progression. But patients with chronic MS pain had independently significantly higher all-cause mortality than those patients without chronic MS pain. However, the inflammatory marker-hs-CRP was similar between CKD patients with and without chronic MS pain.

Our predialysis CKD patients had a high prevalence of chronic MS pain (53.3%) and our results indicate that male patients, high BMI, and co-morbidity of hyperuricemia, and a Ca × P imbalance were significantly associated with this symptom. However, we were surprised to find that patients with and without chronic MS pain used NSAIDs and Chinese herbal medicines at similar levels, had similar levels of inflammatory markers (hs-CRP), and had similar levels of serum albumin. Our long-term follow-up of about 6 years indicated that patients with chronic MS pain had similar risk of CKD progression as those without chronic MS pain. Pain and inflammation are nearly always associated with each other. Hyper-inflammation was associated with CKD progression [17,18]. The results showed that serum levels of hs-CRP were significantly associated with composite endpoints. However, those patients with and without chronic MS pain had similar serum levels of hs-CRP. These results suggested that those CKD patients with chronic MS pain patients only had more local inflammation but did not have more systemic inflammation than those without chronic MS pain patients. This result may be also due to the similar prevalence of baseline NSAID use in patients with and without chronic MS pain. The study showed that those patients used a NSAID at baseline also had similar risk of CKD progression. Previous research indicated that NSAIDs are associated with acute kidney injury in the general population and with disease progression in patients with CKD [19,20]. The NSAID-mediated decrease in volume of renal blood flow, which results from decreased prostaglandin synthesis, can lead to acute kidney injury, sodium retention, edema, hypertension, and hyperkalemia in CKD patients [21]. In addition, habitual NSAID use can also lead to analgesic nephropathy, a condition that is often irreversible upon drug discontinuation [22]. However, in our CKD patients, those with baseline use of an NSAID had similar risk of CKD progression. The reason for the lack association between use of a NSAID drug at baseline and CKD progression may be related to our CKD education program. In the CKD center and also outpatient clinic at the Nephrology department, patients were educated about avoid using nephrotoxic agents such as NSAID or Chinese herbal medicine. Another explanation maybe those patients with chronic MS pain had less co-morbidity and better status of health. However, we adjusted for these factors in our multivariate analysis, and found that the presence of chronic MS pain was not significantly and independently associated with risk of CKD progression.

In this study, we found that CKD stages were not significantly different between chronic MS pain and no chronic MS pain (*p* values=.074). One study showed that chronic MS pain was prevalence in advanced CKD patients (CKD stage 4–5 pre-dialysis) [14]. Because we enrolled CKD patients from stage 1 to 5, we also re-analyzed the association between chronic MS pain and advanced CKD. We also found that there was no significant association between chronic MS pain and advanced CKD (*p* values=.377). So, the result suggested that CKD stages and also advanced CKD were not associated with chronic MS pain. Caravaca et al. enrolled advanced CKD patients and found chronic MS pain was associated with higher inflammation and poor prognosis, but the association was not significant after adjustment of the demographic factor. In our study patients, we found chronic MS pain was significantly associated with all-cause mortality after adjustment. But we cannot find the association between chronic MS pain and inflammation. It may be due to chronic MS pain in some patients may not link to inflammation. Chronic MS pain caused by inflammation is more likely to be associated with advanced CKD patients. Due to the recruited patients, CKD with different stages and the number of our advanced CKD patients was not enough, we cannot find this association in our study patients.

The focus of this study was to examine CKD patients with chronic MS pain and CKD progression. We found that those with chronic MS pain and baseline use of a NSAID or Chinese herbal medicine had similar risk of CKD progression as those without chronic MS pain and use of a NSAID or Chinese herbal medicine. Besides, the inflammatory marker-hs-CRP serum levels were similar between those CKD patients with and without chronic MS pain. Our study has some limitation. The use of NSAID and Chinese herb agents was based on a self-reported questionnaire and the prevalence of NSAID and Chinese herb use was not recorded during the follow-up period. Besides, we did not collect acute kidney injury outcome data. Also, we did not collect the information on opioid use. Opioid is related to proteinuria. Those patients with chronic MS pain may use opioid instead of NSAID or CHM for pain relief. Due to lacking information about opioid use, the study cannot conclude NSAIDs or Chinese herb agents do not associate with CKD progression. In addition, a sample size of NSAIDs or CHM user is too low to detect this association. However, our results found the presence of chronic MS pain was independently significantly associated with increased all-cause mortality.

In summary, our study demonstrated that CKD patients with chronic MS pain were independently and significantly associated with all-cause mortality, but not independently and significantly associated with CKD progression and composite endpoints. The inflammatory marker-hs-CRP was similar between CKD patients with and without chronic MS pain.
